# Characterization of the Differential Adverse Event Rates by Race/Ethnicity Groups for HPV Vaccine by Integrating Data From Different Sources

**DOI:** 10.3389/fphar.2018.00539

**Published:** 2018-05-24

**Authors:** Jing Huang, Jingcheng Du, Rui Duan, Xinyuan Zhang, Cui Tao, Yong Chen

**Affiliations:** ^1^Department of Biostatistics, Epidemiology, and Informatics, University of Pennsylvania, Philadelphia, PA, United States; ^2^School of Biomedical Informatics, University of Texas Health Science Center, Houston, TX, United States

**Keywords:** data integration, HPV, race/ethnicity, vaccine adverse event reporting system, vaccine safety

## Abstract

Data from the Vaccine Adverse Event Reporting System (VAERS) contain spontaneously reported adverse events (AEs) from the public. It has been a major data source for detecting AEs and monitoring vaccine safety. As one major limitation of spontaneous surveillance systems, the VAERS reports by themselves sometimes do not provide enough information to answer certain research questions. For example, patient level demographics are very limited in VAERS due to the protection of patient privacy, such that investigation of differential AE rates across race/ethnicity groups cannot be conducted using VAERS data only. For many vaccines, racial and ethnical difference in immune responses has been found in studies based on racially diverse cohorts. It is of great interest to characterize the differential AE rates by race and ethnicity groups for vaccines. In this study, we propose a novel statistical method to integrate VAERS data with data from other resources for vaccine pharmacovigilance research. Specifically, we integrate VAERS data with CDC survey data of vaccine coverage and U.S. census data of race/ethnicity distribution to quantify differential AE rates by race/ethnicity groups for HPV vaccine. We utilize the difference of race/ethnicity distributions across U.S. states to investigate the association between AE reporting rate and race/ethnicity groups at the population level. We identify 9 AEs with significantly different reporting rates between non-Hispanic White females and other race/ethnicity groups.

## Introduction

Human papillomavirus (HPV) is a known cause of various types of cancers, including cervical cancers, some vulvar, vaginal, anal cancers, etc. (Walboomers et al., 1999; De Vuyst et al., 2009; zur Hausen, 2009). According to the U.S. Center for Disease Control and Prevention (CDC), an average of 38,793 HPV-associated cancers were diagnosed annually during 2008–2012 (Viens, 2016). Since introduced in 2006, HPV vaccines have been used to protect the human against most of the cancers caused by HPV infections (Stokley et al., 2014). Despite recommendations in the U.S. for routine HPV vaccination, rates of HPV vaccine coverage continue to be low (Kester et al., 2013). Safety is considered as one of the major concerns for non-vaccination (Zimet et al., 2010; Kester et al., 2013; Holman et al., 2014; Du et al., 2017b). Common adverse events (AEs) following HPV vaccination are usually mild, but on very rare occasions severe reactions may occur after HPV vaccination (U.S. CDC, 2017). As some AEs are unlikely to be detected in pre-licensure clinical trials due to several reasons including the low frequencies of the AEs, the limited number of enrolled subjects, and the limited follow-up time period, the post-marketing monitoring of AEs from analyzing spontaneously reported AEs is essential (Zhou et al., 2003).

Vaccine Adverse Event Reporting System (VAERS) is a spontaneous surveillance system to accept reports of suspected AEs following any U.S. licensed vaccines (Zhou et al., 2003). VAERS collects information on the age, sex, administered vaccine names, post vaccination events/symptoms, medical history, and etc. (Moro et al., 2018). As a passive surveillance system, VAERS has multiple limitations including underreporting, recall bias, reporting errors, and lack of denominator data and unbiased control groups (Ellenberg and Chen, 1997). Due to these inherent limitations, VAERS data are primarily used for generation of hypothesis for vaccine safety (Centers for Disease Control and Prevention, 2017). Signals detected from VAERS data must be interpreted with caution and need to be further investigated. Despite these limitations, VAERS contributes to public health in critical ways (Chen et al., 1994; Shimabukuro et al., 2015). Due to the increasing national coverage, VAERS collects national data from all U.S. states and territories and provides a unique opportunity in monitoring vaccine AEs that might occur too rarely to be detected in pre-licensure clinical trials or even post-marketing active surveillance programs (Ellenberg and Chen, 1997). It serves as an early warning system and guide regulators to conduct controlled research into various safety questions. VAERS data can be used to reveal possible indications on risk predication of a reaction following vaccination. Therefore, Effective analysis of VAERS data is vital to assure the safety of vaccines.

Many efforts have been done to investigate AEs following HPV vaccination using VAERS data in recent years. Geier et al. evaluated the risk of serious autoimmune adverse events following HPV vaccination (Geier and Geier, 2017). Arana et al. reviewed postural orthostatic tachycardia syndrome after HPV vaccination (Arana et al., 2017). Slade et al. provided a summary of VAERS reports for HPV vaccine including reporting rates of AEs following HPV vaccine, and compared AEs with expected background rates (Slade et al., 2009). However, to our best knowledge, no current effort has investigated the individual differences of experiencing AEs after HPV vaccination. Individual characteristics, e.g., age, gender, race, can have impacts on the immune responses to a vaccine (Poland et al., 2009). Our previous studies have identified individual differences (age and gender) in adverse reactions following trivalent influenza vaccination using VAERS data (Du et al., 2016, 2017a). Racial and ethnical difference in immune responses has also been found in different vaccines from studies based on racially diverse cohorts, including rubella vaccination (Haralambieva et al., 2014), influenza vaccination (Gardner et al., 2006). As VAERS does not collect racial and ethnical information due to privacy consideration, it creates a roadblock on the analysis of race/ethnicity difference in reported AEs following HPV vaccinations. In order to utilize the valuable information of AEs reports in VAERS for such analyses, it is necessary to combine VAERS data with additional data collected from other sources that can provide racial/ethnical information of vaccine recipients. To this end, novel statistical methods that can effectively integrate data from multiple sources are needed.

In this paper, we fill the research gap by proposing a novel statistical method to integrate VAERS data with data from other resources for vaccine pharmacovigilance research. Specifically, using a probability model, we combine the VAERS data with CDC national survey data of vaccination coverage and U.S. census data of race/ethnicity distribution in different states to investigate the differential AE rates in different race/ethnicity groups. To our best knowledge, this is the first attempt to study racial/ethnical difference following vaccination using VAERS data. Specifically, we combine aggregate data at the U.S. state level to tackle the challenge of unavailability of individual level racial/ethnical data. Such a formulation also has the advantage of preserving individual's privacy. Specifically, our method uses a generalized linear mixed effects model to link three sources of datasets, where the components of the model are approximated by different data sources. The random effects of the model account for the correlation of data collected from the same state at different years, and allow the heterogeneity of HPV vaccine coverage and number of AE reports following HPV vaccine across states. We illustrate our method to compare the reporting rate of AEs after HPV vaccination in non-Hispanic White females vs. other race/ethnicity groups. When fitting the model, we also consider the feature of large number of zero observations in VAERS by fitting a zero-inflated model. In our analyses, we identify 5 AEs which were reported with higher rates in non-Hispanic White females, and 4 AEs were reported with lowers rates in non-Hispanic White females.

## Methods

Due to the unavailability of individual level race/ethnicity data in VAERS, current methods that can be used to analyze the differential rates of reporting AEs in different race/ethnicity groups using VAERS reports are scarce. We propose a method to answer such a question by combining data from VAERS reports, CDC national survey of vaccination coverage, and U.S. census of race/ethnicity distribution in the 50 U.S. states. Specifically, our method considers individual state as the study unit, and utilize the heterogeneity of number of VAERS reports for a given AE across states and the heterogeneity of race/ethnicity distribution across states to investigate whether the difference in the reporting rate of the AE across states can be partially explained by the difference in the race/ethnicity distribution. In addition, the proposed method uses CDC survey data and U.S. census data to adjust for the total number of vaccinated population at each state. Integration of the three data sources is through a generalized linear mixed effects model, where the components of the model are approximated by different data sources. The random effects allow the heterogeneity of HPV vaccine coverage and number of AE reports following HPV vaccination across states and account for the with-state correlation of data collected from different years. We will introduce the model and illustrate the proposed method using the VAERS reports for HPV vaccine. The proposed method can be used to study other vaccines as well.

(1) $$\begin{array}{l} x_{ij}=\frac{\text{total numml:mber of non-Hispanic White femml:males who received HPV vaccine in the i-th state at the j-th year}}{\text{total numml:mber of people who received HPV vaccine in in the i-th state at the j-th year}}\\ =\frac{\begin{array}{l} \text{total numml:mber of non-Hispanic White femml:males in the i-th state at the j-th }{\text{year}}^{*}\text{HPV vaccine coverage amml:mong non}\\ \text{-Hispanic White femml:males in the i-th state at the j-th year } \end{array}}{\text{total numml:mber of people who received HPV vaccine in the i-th state at the j-th year}}\\ =\frac{z_{ij}\;w_{ij}\;}{V_{ij}} \end{array}$$

### Probability model

For a given AE, consider the research question “whether the reporting rate of the AE is the same in the non-Hispanic White female population comparing to the other race/ethnicity population.” Let ***n***_**ij**_, ***y***_**ij**_, and ***x***_**ij**_ denote the total number of HPV vaccine recipients, the total number of VAERS reports of the AE, and the percentage of non-Hispanic White females in HPV vaccine recipients in the i-th state at the j-th year, *i* = 1,…,50, *j* = 1,… J, where J is the total number of years observed. Given the total number of HPV vaccine recipients, the total number of VAERS reports of the AE in the i-th state at the j-th year can be assumed to follow a binomial distribution, i.e., ***y***_***ij***_ **~ *BN*(*n***_***ij***_**, *p***_***ij***_**)**, where ***p***_***ij***_ is the rate of reporting the AE in the i-th state at the j-th year. Since we are interested in studying severe AEs, which are often rare events, the binomial distribution can be approximated using a poisson model such that

(2) $$\begin{array}{r} y_{ij}\;\sim \;Poisson\;\left(n_{ij}p_{ij}\right), \end{array}$$

where *n*_*ij*_*p*_*ij*_ is the expected number of events in the i-th state at the j-th year. Considering that data from the same state can be correlated across years, we will use a generalized linear mixed model to pool data from different years. Specifically, to study whether the reporting rate of the AE is the same in the non-Hispanic White female population comparing to the other race/ethnicity population, or equivalently whether the difference of reporting the AE across states can be partially explained by the difference in percentage of non-Hispanic White females in HPV vaccine recipients across states, a generalized linear mixed model can be assumed as follows:

(3) $$\log\{E(y_{ij}|x_{ij})\}=\;\beta_{0i}+\;\beta_{1}\log(x_{ij})+log(n_{ij}),$$

where the fixed effect β_1_ quantifies the association between race/ethnicity (percentage of non-Hispanic White females in HPV vaccine recipients) and the occurrence of AE following HPV vaccination at the log scale, the random intercept β_0*i*_ accounts for the correlation of observed number of events for the i-th state across years and quantifies the baseline risk of observing the AE among people who received HPV vaccination and are not non-Hispanic White females, and log(*n*_*ij*_) is the offset to adjust for the total number of people who received HPV vaccination in the i-th state at the j-th year.

In practice, data of *x*_***ij***_ are difficult to obtain. However, with some linear algebra, *x*_***ij***_ can be calculated as follows:

Plugging equation (3) to equation (2), we get.

(4) $$\begin{array}{r} \log\{E(y_{ij}|z_{ij},w_{ij})\}=\;\delta_{0i}+\;\beta_{1}\log(z_{ij}w_{ij})+\log(n_{ij}), \end{array}$$

where δ_0*i*_ = β_0*i*_+β_1_log(*V*_*ij*_), and *z*_***ij***_, *w*_***ij***_, and *V*_***ij***_ are the total number of non-Hispanic White females, the HPV vaccine coverage (percentage of HPV vaccine recipients) among non-Hispanic White females, and the total number of people who received HPV vaccination in the i-th state at the j-th year, respectively. After the transformation, the data of **y**_***ij***_, *w*_*ij*_, and *z*_*ij*_ can be obtained from VAERS reports, the CDC survey of vaccination coverage and the data from the U.S. census, respectively. With such a formulation, we are able to study the association between race/ethnicity (non-Hispanic White female vs. others) and the occurrence of AE after HPV vaccination by estimating β_1_, and answer the question “whether the reporting rate of the AE is the same in the non-Hispanic White female population comparing to the other race/ethnicity population” by testing null hypothesis: β_1_ = 0. In addition, our formulation does not require data of the total number of people who received HPV vaccination in the i-th state at the j-th year, *V*_*ij*_, which are often difficult to obtain in reality. Instead, β_1_log(*V*_*ij*_), which is not of the primary interests, is formulated as a component of the random intercepts, and is not estimated separately.

### Data sources

#### VAERS

We searched VAERS data for U.S. reports submitted from 2010 to 2016 after the receipt of the following HPV vaccines: HPVX (Human papillomavirus, no brand name), HPV2 (human papillomavirus 2-valent), HPV4 (human papillomavirus 4-valent), and HPV9 (human papillomavirus 9-valent). We then extracted the serious reports with one of the serious AE outcomes (i.e., death, life-threatening illness, hospitalization, prolonged hospitalization, or permanent disability) (Roush et al., 2008).

#### CDC survey

Data of the HPV vaccination coverage among adolescents aged 13–17 years from year 2010 to 2016 were acquired from National Immunization Survey-Teen (NIS-Teen). NIS-Teen monitors vaccination coverage among adolescents aged 13–17 years in the 50 states using a random-digit–dialed sample of landline and cell phone numbers (Reagan-Steiner, 2016). NIS-Teen collects race/ethnicity information reported by parent/guardian respondent and reports the coverage of HPV vaccine among different racial and ethnicity groups in different states each year. We extracted the coverage data of at least one dose of HPV vaccination among two racial and ethnicity groups: non-Hispanic White females and others.

#### U.S. census

The percentage of non-Hispanic White females from year 2010 to 2016 in each states were obtained from the U.S. Census Bureau's annual estimates of the resident population by sex, race, and Hispanic origin for the United States from April 1, 2010 to July 1, 2016, which were based on the 2010 Census and reflect changes to the April 1, 2010 population (U. S. Census Bureau, 2017).

### Data analysis

Upon searching the VAERS for HPV vaccine in the United States from 2010 to 2016, we extracted reports with serious AEs as defined above. All of these reports were manually coded using Medical Dictionary for Regulatory Activities (MedDRA) vocabulary by CDC/FDA (ICH, 2017). The AEs were coded as preferred terms (PTs) in MedDRA. Each PT is “a distinct descriptor (single medical concept) for a symptom, sign, disease diagnosis, therapeutic indication, investigation, surgical, or medical procedure, and medical social or family history characteristic” (MedDRA, 2017). The selected VAERS reports were transformed to a dataset containing the total number of events reported for each observed PT by state and year. Since most of the PTs were serious and rare events, the data contain a large number of zero-counted cells. Considering such a feature, we conducted zero-inflated poisson regression analysis with mixed effects using R function glmmTMB from R package glmmTMB.

## Results

### Data visualization

**Figure 1:**
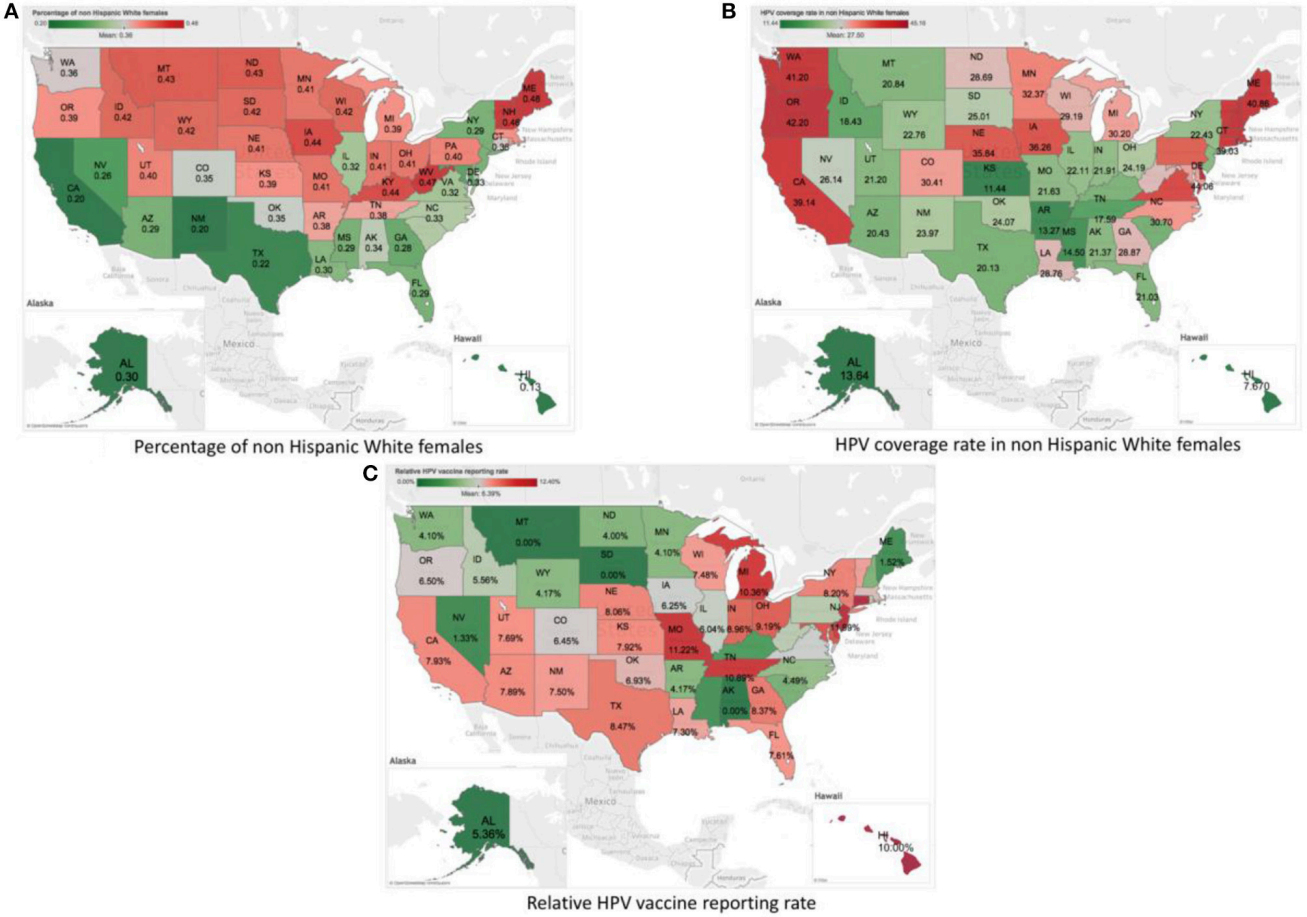
Visualization of the summary statistics from the three data sources by States using the U.S. map. **(A)** Plot of percent of non-Hispanic White females by Sates according to the U.S. census data. **(B)** Plot of HPV vaccine coverage rate among non-Hispanic White females by States based on the CDC survey data of HPV vaccine coverage. **(C)** Plot of the percentage of HPV-vaccine-related VAERS reports among all the VAERS reports by States based on the VAERS data.

Data extracted from the VAERS reports for HPV vaccine from 2010 to 2016 included 37,271 reports. 1,843 out of these reports were identified as serious. 1,397 of them include the location information to the U.S. States level. We then limited the reports with the age to adolescents 13–17 years, which resulted in 355 reports with 2,864 unique PTs. We organized the data in a format with each row representing one of the 50 states at one of the 7 years, the dataset for analysis is a 350 × 2,864 table with 993,409 zero-count cells (99.2%). Data from the CDC HPV vaccine survey and the U.S. census were organized in the similar way. A visualization of the three data sets are shown in Figure 1. Figure 1A shows the percentage of non-Hispanic White females in each state according to the U.S. census data. The the national average percentage is showed in gray. States with values higher than the national average are showed in red, and states with values lower than the national average are showed in green. Darker colors indicate values farther away from the national average. We can see that states in the southwestern coast area tend to have a lower rate of non-Hispanic White females. Figure 1B shows the coverage of HPV vaccination among non-Hispanic White females in different states based on the CDC survey data of HPV vaccine coverage. The national average of HPV vaccination coverage among non-Hispanic White females is around 27.5%. We found that the coverage of HPV vaccination among non-Hispanic White females is relatively higher in the western and eastern coast areas (up to 42.2% in Oregon), and lower in the middle and south of the U.S. (with lowest around 11.4% in Kansas). We also calculated the percentage of VAERS reports for HPV vaccine among all the VAERS reports. The results were shown in Figure 1C. The national average percentage of VAERS reports for HPV vaccine among all the VAERS reports is around 6.4%. We found that the percentage is relatively higher in states in the south area, e.g., 7.9% in Kansas and 11.2% in Missouri.

### Differential adverse event rates for HPV vaccination between race/ethnicity groups

**Figure 2:**
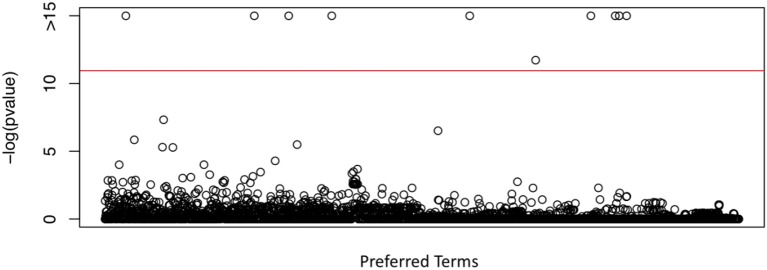
*P*-values obtained from the proposed test in testing the differential PT rates for HPV vaccination between non-Hispanic White female and other race/ethnicity group, using VAERS data, CDC survey data of HPV vaccination coverage and U.S. census data.

We investigated the association of the reporting rate of each PT with the percentage of non-Hispanic White female HPV vaccine recipients using the proposed mixed effects zero-inflated poisson regression analysis. Among the 2,864 PTs, we identified ten PTs, with Bonferroni correction, which have significantly different reporting rates when the percentage of non-Hispanic White female HPV vaccine recipients is different across states. They are “vulvovaginal rash,” “lichen sclerosus,” “vulvovaginal pain,” “electrocardiogram change,” “fecal incontinence,” “pulse absent,” “bedridden,” “respiration abnormal,” “blood electrolytes normal,” and “tension headache.” The PT “blood electrolytes normal” indicates a normal result of an investigation procedure rather than an AE, so we excluded it in our investigation. The *p*-values of all 2,864 PTs were shown in Figure 2. The red line indicates the significant level of 0.05 after Bonferroni correction.

**Figure 3:**
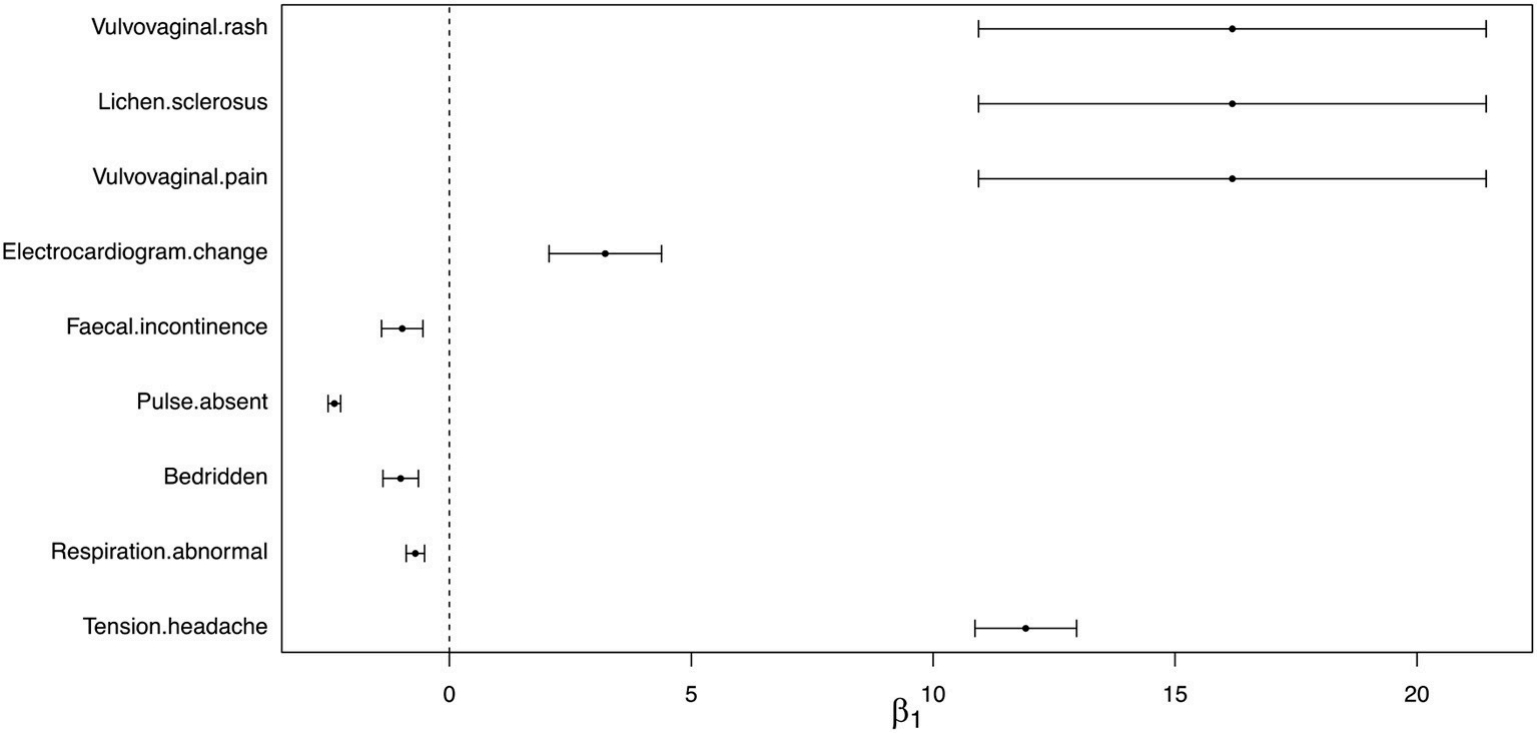
Estimated effect size and 95% confidence interval of reporting AEs between non-Hispanic White female and other race/ethnicity group.

For the 9 identified AEs, we further estimated the effect size of being non-Hispanic White female HPV vaccine recipients on the risk of observing these AEs. The effect size estimates and 95% confidence intervals were shown in Figure 3. We found that with higher percentage of non-Hispanic White female HPV vaccine recipients, the rates of observing “fecal incontinence,”“pulse absent,” “bedridden,” and “respiration abnormal” decreased, and the rates of observing “vulvovaginal pain,” “vulvovaginal rash,” “lichen sclerosus,” “electrocardiogram change,” and “tension headache” increased.

## Discussion

VAERS and other post-marketing surveillance data usually contain a large number of information about AEs but relatively less information on patient demographics. Study of the VAERS data alone can only answer a limited number of research questions. In this paper, we proposed a statistical approach to integrate VAERS data with CDC national survey data and U.S. census data to investigate the differential risk of experiencing AEs after vaccination in different race/ethnicity groups. By combining VAERS data with data from different sources, the proposed method increased the utility of the VAERS database to answer more complex research questions for vaccine safety. To our best knowledge, our method is the first to integrate VAERS data with data from other sources to study the association between AEs and race/ethnicity for vaccine safety. We illustrated the method using HPV vaccine, and we identified 9 AEs with significantly different reporting rates between the non-Hispanic White females and other race/ethnicity group after HPV vaccination. The proposed method is a general tool, which can be used to detect AEs that have different reporting rates in different race groups for different vaccines. In our investigation, the choice of studying non-Hispanic White females (vs. the rest) is due to the reason that the CDC survey does not contain enough data for other race/ethnicity groups for HPV vaccine. We will apply our method to comparisons of other races/ethnicity groups, when this information becomes available.

The major strength of the proposed method is that the data integration is through a generalized linear mixed effects model, where information from different data sources are combined effectively as different components of the model. Specifically, the random effects account for the unique features of the data, by allowing the heterogeneity of data across states and accounting for the with-state correlation across years. Moreover, the proposed method also accounts for the large number of zero observations in VAERS by using zero-inflated Poisson model in the analysis. Secondly, the proposed method integrates data at the aggregate data level, i.e. data are aggregated by state, which addresses the limitation that individual level demographic information are often unavailable or limited in VAERS reports and has the advantage of privacy protection. The proposed method adopts a similar philosophy from the meta-regression analysis, which utilizes the heterogeneity of race/ethnicity distribution across states and the heterogeneity of reporting rates of AE across states to study “whether the heterogeneity of reporting rate can be explained by the difference in percentage of vaccinated non-Hispanic White females across states.” Rejection of such a null hypothesis implies that there are differential risks of experiencing AE at different race groups after HPV vaccination. In addition, the proposed method also addresses the challenge of unavailability of the total number of HPV vaccinated population by a transformation of the probability model and a formulation of the unknown data as a component of the random intercepts in the model.

Our investigation also has a few limitations. First, the differences in HPV vaccine types (e.g., HPV2, HPV4, and HPV9) and manufactures could be partially impactful on AEs following vaccination. However, in our study, we did not consider such differentiations, due to the reason that the sample size in each category of HPV vaccine can be too small to gain any statistical power. Secondly, the age range of the three data sets were not perfectly aligned. Both the CDC data of HPV coverage and the VAERS data were collected for adolescents with 13–17 years old, but the U.S. census data of racial/ethnical distribution were not. It can be a potential problem if the percentage of non-Hispanic White females between 13 and 17 residents are substantially different from the overall percentage among all the residents. Thirdly, we assume the same reporting rate for different racial/ethnical population groups in VAERS. However, this may not be true as the data of reporting rates can not be acquired from VAERS. In addition, due to the intrinsic limitation of VAERS, the association identified in our study cannot be interpreted as causality. Further investigation with more sophisticated study design are needed to confirm the 9 AEs that were observed with differential reporting rates at different race/ethnicity groups after HPV vaccination.

As of most studies based on observational data, potential observation errors (e.g., measurement errors, misclassifications) of data from each of the databases may bias the findings. The mechanisms of measurement errors or misclassifications of observations are many, including differential and non-differential. If the probability model of the measurement error or misclassification is the same for all individuals, it is called non-differential measurement error or misclassification. Otherwise, it is differential measurement error or misclassification. It has been shown that, under non-differential measurement error or misclassification, the point estimate of the association is biased toward the null, named as “attenuation,” which will not lead to inflation/deflation of Type I errors (Neuhaus, 1999). In the proposed method, we focused on hypothesis testing to identify the safety signals. The results are valid if the measurement errors in the databases are non-differential. We are extending the proposed method to account for more complex measurement errors, such as differential misclassifications.

In our study of the HPV vaccine data using the proposed method, we identified 9 AEs with significantly different reporting rates between the non-Hispanic White and the others. The AEs “vulvovaginal rash,” “lichen sclerosus,” and “vulvovaginal pain,” which may be related to autoimmune disorders, were found to have higher rates in non-Hispanic White females than the others. Such a potential association between HPV vaccination and autoimmune disorders were also investigated and reported in current literature, but the evidence for the association were not consistent. Cases of autoimmune disorder following HPV vaccination were reported(Colafrancesco et al., 2013; Tomljenovic et al., 2014; Palmieri et al., 2017), while some studies also found statistically insignificant association (Arnheim-Dahlström et al., 2013; Hviid et al., 2018). Such inconsistent findings can be potentially due to differential rates of AE among race and ethnicity groups, but to our knowledge, no current study has investigated differential AE following HPV vaccination among race and ethnicity groups, although differential rates of uptaking the vaccine among race and ethnicity groups has been reported (Bednarczyk et al., 2011), with a lower rate in black/African-American women compared to White. Recent studies also reported the increased rate of Guillain-Barré syndrome (GBS) and incidence of neurological disorder related AEs following HPV vaccination (Ikeda, 2015; Miranda et al., 2017). In our study, we also identified three AEs that are potentially related to neurological disorders: “fecal incontinence,” “respiration abnormal,” and “tension headache.” In particular, these three AEs are found to have lower rates in non-Hispanic White females than the others. In a recent review of AEs after HPV vaccination (Martínez-Lavín and Amezcua-Guerra, 2017), it was shown that both existing pre-licensure randomized trials and post-marketing studies and reports identified similar AE symptoms, including headache, fatigue, dizziness, musculoskeletal pain, and gastrointestinal symptoms among others. In our investigation, we also found the rate of pulse absent, which is potentially related to cardiovascular disorders, is higher in non-Hispanic White females compared to the others.

In our investigation, we observed that the HPV vaccine coverage is different across states, which can be a potential indication of health disparity caused by state policy, financial support and income status. While CDC has made many efforts to increase HPV vaccine use, it is also important to investigate the effect of potential risk factors for health disparity of HPV vaccination in the future.

## Conclusion

By adopting a generalized linear mixed effects model, we integrated VAERS data with CDC national survey data and U.S. census data to investigate the differential risk of AEs following vaccination between non-Hispanic White females and other race/ethnicity group. Our method was the first effort to combine data from other sources with VAERS data to study the association between AE rate and race/ethnicity group for vaccine safety. Through the integration of information from different databases, the proposed method increased the utility of the VAERS database to answer more complex research questions for vaccine safety.

The safety of HPV vaccination is an important public health issue. The association between safety of HPV vaccination and race/ethnicity had not been extensively studied in literature. In our investigation, we found 9 AEs with significantly different reporting rates following HPV vaccination between the non-Hispanic White females and other race/ethnicity group. Considering the importance of HPV vaccination, these signals warranted further investigations.

## Author contributions

YC, CT, JH, and JD proposed the methods and experiments. YC and JH formulated the model. JH and JD cleaned the data, conducted the data analyses. JH, JD, RD, XZ, CT, and YC interpreted the results and provided instructive comments. JH and JD drafted the main manuscript. All the authors read and approved the final version of the manuscript.

### Conflict of interest statement

The authors declare that the research was conducted in the absence of any commercial or financial relationships that could be construed as a potential conflict of interest.
